# Supplementary material for: Dynalign II: common secondary structure prediction for RNA homologs with domain insertions
Source: Nucleic Acids Res. 2014 Nov 21;42(22):13939–48. doi: 10.1093/nar/gku1172 (PMC4267632; doi:10.1093/nar/gku1172)
Supplement: SUPPLEMENTARY DATA [file supp_gku1172_nar-02021-z-2014-File012.zip › manual/GUI/html/Stochastic_Sampling.html]

RNAstructure GUI Help -- Stochastic Sampling


|  |  |  |
| --- | --- | --- |
|  | RNAstructure GUI Help Stochastic Sampling | - Contents - Index |
| The stochastic sampling module generates a sample of secondary structures, with the probability of choosing a secondary structure equal to its probability of occurrence in the complete folding ensemble.  **How to Predict a Stochastic Sample**   1. Do a partition function calculation with the sequence of interest. (See "Partition Function.") Any folding constraints must be applied at the partition function calculation step. Also, any changes in the folding temperature must be applied at the partition function step. 2. Start the module by choosing "Stochastic Sampling." This module is available for both RNA and DNA. 3. Click the button labeled "Partition Function Save File" to choose a save file for use in sampling. 4. The default value of the output file, which is filled in after save file selection, can be changed by clicking the "CT File" button. 5. The default ensemble size and random seed can also be changed. Changing the random seed changes the sample of structures generated. 6. Click "Start" to run the calculation. A progress bar will open to show the progress of the sampling. 7. When the calculation is complete, the option is given to draw the set of structures sampled. Click "OK" to draw the structures or "Cancel" to exit the module. | | |
| Visit The Mathews Lab RNAstructure Page for updates and latest information. | | |
